# Enhancement of the haemostatic effect of platelets in the presence of high normal concentrations of von Willebrand factor for critically ill patients needing platelet transfusion—a protocol for the will-plate randomised controlled trial

**DOI:** 10.1186/s13063-022-06876-8

**Published:** 2023-01-20

**Authors:** Goetz Herrmann, Andrea Blum, Daniel Bolliger, Rita Achermann, Anna Estermann, Caroline Eva Gebhard, Anne Henn, Jan Huber, Jasprit Singh, Atanas Todorov, Tatjana Zehnder, Núria Zellweger, Andreas Buser, Dimitrios A. Tsakiris, Alexa Hollinger, Martin Siegemund

**Affiliations:** 1 Department for Anesthesia, Prehospital Emergency Medicine and Pain Therapy, University Hospital Basel, Spitalstrasse 21, 4031 Basel, Switzerland; 2 Intensive Care Unit, University Hospital Basel, Spitalstrasse 21, CH-4031 Basel, Switzerland; 3 Medical Faculty of the University of Basel, Klingelbergstrasse 61, 4056 Basel, Switzerland; 4 Hospital Pharmacy, University Hospital Basel, Spitalstrasse 26, 4031 Basel, Switzerland; 5 Department of Nuclear Medicine, Cardiovascular Gender Medicine, University Hospital Zurich, Rämistrasse 100, 8091 Zurich, Switzerland; 6 Transfusion Medicine and Regional Blood Transfusion Service Swiss Red Cross, Department of Hematology, University Hospital of Basel, Petersgraben 4, 4031 Basel, Switzerland; 7 Department for Diagnostic Haematology, University Hospital of Basel, Petersgraben 4, 4031 Basel, Switzerland

**Keywords:** Adult intensive and critical care, Blood loss, Randomised controlled trial, VerifyNow, von Willebrand factor

## Abstract

**Introduction:**

von Willebrand Factor (vWF) is a key protein mediating platelet adhesion on the surface of damaged endothelia. To the best of our knowledge, no trial exists that investigated the effect of platelet transfusion in combination with the administration of balanced vWF in severe blood loss, despite being widely used in clinical practice. The Basel Will-Plate study will investigate the impact of the timely administration of balanced vWF (1:1 vWF and FVIII) in addition to platelet transfusion on the need for blood and coagulation factor transfusion in patients admitted to the intensive care unit (ICU) who suffer from severe bleeding. The study hypothesis is based on the assumption that adding balanced vWF to platelets will reduce the overall need for transfusion of blood products compared to the transfusion of platelets alone.

**Methods and analysis:**

The Will-Plate study is an investigator-initiated, single-centre, double-blinded randomised controlled clinical trial in 120 critically ill patients needing platelet transfusion. The primary outcome measure will be the number of fresh frozen plasma (FFP) and red blood cell (RBC) transfusions according to groups. Secondary outcome measures include the number of platelet concentrates transfused within the first 48 h after treatment of study medication, quantity of blood loss in the first 48 h after treatment with the study medication, length of stay in ICU and hospital, number of revision surgeries for haemorrhage control, ICU mortality, hospital mortality, 30-day mortality and 1-year mortality. Patients will be followed after 30 days and 1 year for activities of daily living and mortality assessment. The sample size was calculated to detect a 50% reduction in the number of blood products subsequently transfused within 2 days in patients with Wilate® compared to placebo.

**Ethics and dissemination:**

This study has been approved by the Ethics Committee of Northwestern and Central Switzerland and will be conducted in compliance with the protocol, the current version of the Declaration of Helsinki, the ICH-GCP or ISO EN 14155 (as far as applicable) and all national legal and regulatory requirements. The study results will be presented at international conferences and published in a peer-reviewed journal.

**Trials registration:**

ClinicalTrials.gov NCT04555785. Protocol version: Clinical Study Protocol Version 2, 01.11.2020. Registered on Sept. 21, 2020.

**Supplementary Information:**

The online version contains supplementary material available at 10.1186/s13063-022-06876-8.

## Strengths and limitations of the study

- The study’s main strength is the implementation of a promising and secure therapy approach for patients suffering from severe bleeding.
- This is a prospective, randomised, double-blind, placebo-controlled clinical trial for data of high-quality evidence.
- The study is limited by the heterogeneity of critically ill patients and therefore the underlying causes of bleeding. However, bleeding sources will be documented.

## Background and rationale

Von Willebrand factor (vWF) represents a key protein produced by the endothelial cells as a heterogeneous mixture of low- and high-molecular-weight units. vWF is a ligand for receptors on the platelet surface and endothelial cells (GPIb-V-IX, αIIbβ3, αvβ3) mediating adhesion of platelets to each other or to the surface of damaged endothelium and supporting platelet activation. Initial platelet adhesion is a crucial step in haemostatic function [1, 2]. It plays also an important role in protecting FVIII from early activation and clearance^1^.

Several vWF preparations exist (e.g. Wilate, Hemate-P, Alphanate, Wilfact, Veyvondi), the majority of which are combined with antihaemophilic factor (coagulation factor VIII). The product’s included coagulation factor VIII acts in the activated form like the regular factor VIIIa. It takes part in the coagulation amplification by activating factor X to Xa together with factor IXa. Activation of factor X results in generating thrombin from prothrombin. With the administration of both products—von Willebrand factor and coagulation factor VIII—thrombocyte function and overall coagulation may be improved. Wilate® is a 1:1 balanced mixture of von Willebrand factor and coagulation factor VIII registered in Switzerland for prophylaxis and treatment of bleeding in patients suffering from von Willebrand disease and haemophilia A.

There is suggestive evidence from an in vitro flow chamber model and from the treatment of patients with severe vWF deficiency that increasing the concentration of vWF onto normal or supranormal levels can enhance platelet adhesion independent from platelet count [4]. This might translate into a better haemostatic effect of administered platelet concentrates in the bleeding patient and less need for transfusion of blood products (platelet concentrates), especially in clinical conditions with a high probability of low platelet count and low-vWF activities (e.g. heart surgery with extracorporeal circulation or patients under extracorporal membrane oxygenation (ECMO)).

To the best of our knowledge, no trial exists that investigated the effect of platelet transfusion in combination with the administration of balanced vWF in patients with a need for platelet transfusion, despite being widely used in our clinical practice. With this trial, we will investigate if timely administration of balanced vWF (1:1 vWF and FVIII) in addition to platelet transfusion can increase haemostatic efficacy and reduce the transfusion of allogeneic blood products and the need for additional coagulation factor concentrates.

## Hypothesis

In the randomised Will-Plate study, we aim to test the hypothesis that a single simultaneous transfusion of platelets and balanced vWF concentrate reduces the overall need for transfusion of blood products compared to transfusion of platelets alone (i.e. compared to placebo) with a functional focus on platelet concentrates.

## Methods

### Study design

The Will-Plate study is an investigator-initiated, single-centre, prospective, randomised, double-blind, placebo-controlled clinical trial of patients suffering from severe blood loss.

### Approvals

Approval to conduct this study was granted by the Ethics Committee of North-western and Central Switzerland (EKNZ 2020-02106) in December 2020 and by the Swiss Agency for Therapeutic Products (Swissmedic, 2021DR4037) in March 2021. This study was registered at the Swiss National Clinical Trial Portal (SNCPT; Identifier: SNCTP000004204) and at ClinicalTrials.gov (Identifier: NCT04555785).

### Study setting

Patients from the intensive care unit of the University Hospital of Basel are primarily scheduled for study participation. This is an adult ICU at a Swiss academic tertiary medical centre treating medical or surgical patients.

### Study population

#### Inclusion criteria

Participants fulfilling all of the following inclusion criteria are eligible for the study:

1. Age ≥ 18 years
2. Admission to the intensive care unit (ICU)
3. Patients needing platelet transfusion with or without severe bleeding or prior treatment with single or dual antiplatelet agents (ASS, prasugrel, clopidogrel, ticagrelor)
4. Consent by the patient or a family member (non-emergency cases) in addition to the statement of an independent ICU physician

#### Exclusion criteria

The presence of any one of the following exclusion criteria will lead to exclusion of the participant:

1. Patients receiving factor vWF/VIII concentrate before inclusion in the study (e.g. Haemate®, Wilate®)
2. Women who are pregnant or breastfeeding
3. Participation in another study with an investigational drug within the 30 days preceding and during the present study
4. Overt disseminated intravascular coagulation (DIC)
5. Heparin-induced thrombocytopenia (HIT)
6. Thrombotic thrombocytopenic purpura (TTP) or haemolytic uremic syndrome (HUS)
7. Patients with known inherited thrombocytopathies or inherited coagulation disorders, including known von Willebrand disease or haemophilia A
8. Previous enrolment into the current study
9. Contraindications to the class of drugs under study, e.g. known hypersensitivity or allergy to the class of drugs or the investigational product

## Study period overview

**Fig. 1:**
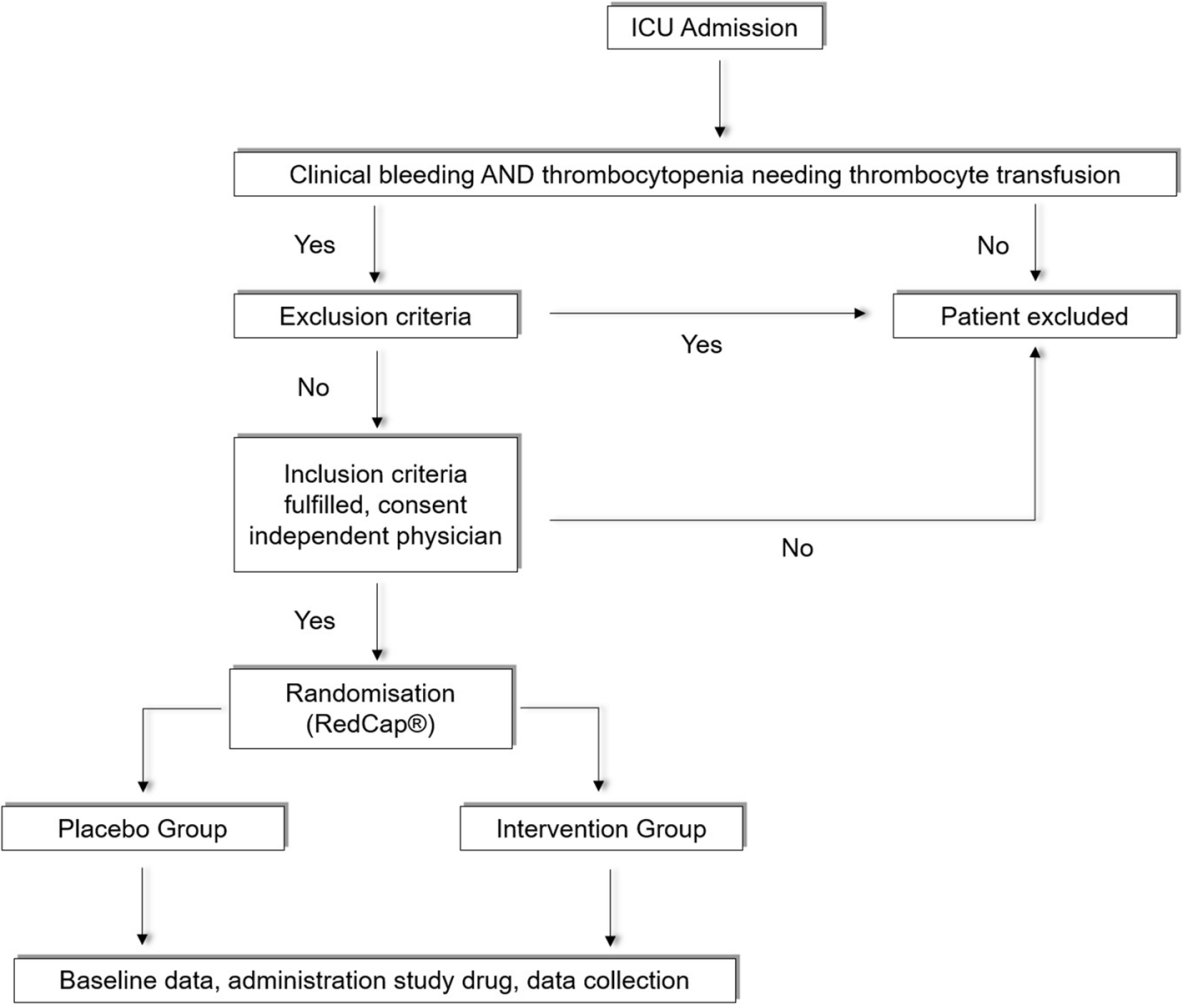
Study flow chart

All patients needing platelet transfusion will be screened for study eligibility (Fig. 1). Upon enrolment, recruited patients will be randomised to one of the two treatment arms (i.e. platelet transfusion with vWF or placebo) with consecutive ICU follow-up for 2 days (Table 1). It will not be allowed to administer another dose of Wilate®/placebo for the study period of 48 h after the first dose was given.

**Table 1:** Study overview

***Visit***	***Screening***	***Treatment***	***Assessment***	***ICU follow-up***		***30-day and 1-year follow-up***^***b***^
*Time (hours, days, weeks)*	*− 1 h*	*0*	*30–60 min*	*1 day*	*2 days*	*12 months*
*Patient information and informed consent*	*x*^*a*^	*x*^*a*^	*x*^*a*^	*x*^*a*^	*x*^*a*^	
*Demographics*	*x*					
*Medical history*	*x*					
*In-/exclusion criteria*	*x*					
*Vital signs*	*x*		*x*	*x*	*x*	
*Platelet function analysis*	*x*		*x*			
*Laboratory tests*	*x*		*x*	*x*	*x*	
*Pregnancy test*	*x*					
*Randomisation*		*x*				
*Information related to bleeding*	*x*		*x*	*x*	*x*	
*Study medication*		*x*				
*Primary outcome variable*			*x*	*x*	*x*	
*Secondary outcome variables*			*x*	*x*	*x*	*x*
*Adverse events*			*x*	*x*	*x*	
*Mortality* *ICU* *Hospital* *30 days* *1 year*				*x*	*x*	*x*

^a^If not possible so far

^b^Assessment of ICU and hospital length of stay, ICU and hospital mortality, 30-day mortality, 1-year mortality, and estimated costs of blood transfusion products

### Screening

Patients will be recruited at the ICU after cardiac surgery, multiple trauma, ECMO or (other) diseases going along acute clinical bleeding at the time of thrombocyte transfusion prescription.

### Thirty-day and 12-month follow-up

Survivors of critical illness not only suffer from long-term disability but also reduced long-term survival [5–7]. To assess the long-term follow-up of patients who received vWF and compare it to those who received placebo, we will perform a follow-up at 30 days and 12 months after the prevailing hospital case has been officially closed (discharge date). With this follow-up, we will assess the following information equally at 30 days and 12 months (information either given by the patient or his/her family/contact person):

- Death after hospital discharge
- Hospital readmission

## Study medication

In case of severe bleeding, the randomised study drug will once be administered. The latter will allow to detect the suggested reduction of the need for blood products.

### Experimental intervention

Wilate® is a 1:1 balanced mixture of von Willebrand factor and coagulation factor VIII. It belongs to the group of anti-haemorrhagic and coagulation factors. It is extracted from plasma, freeze-dried and virus-inactivated by two steps. In Switzerland, it is registered to prevent and treat bleeding of patients suffering from haemophilia A and von Willebrand disease. It is administered as dry solid, which has to be dissolved with the included solvent (water for injection including 0.1% polysorbat 80).

The vWF of the product acts like the vWF of human plasma. It is a key protein mediating platelet adhesion on the surface of damaged endothelial cells or collagen, initiating platelet-platelet aggregation and supporting platelet activation. It plays also an important role in protecting FVIII from early activation and clearance.

The product’s coagulation factor VIII acts in the activated form like the regular factor VIIIa. It takes part in the coagulation amplification by activating factor X to Xa with factor IXa as a co-factor. Activation of factor X generates thrombin from prothrombin.

Wilate® is approved in Switzerland for prophylaxis and treatment of bleeding in patients suffering from von Willebrand disease and haemophilia type A.

In case of haemorrhage and indicated platelet transfusion, a study package will be prepared by an unblinded site member and afterwards provided to the participating patient’s physician in charge with one dose of Wilate® (2000 IU vWF and 2000 IU factor VIII and water for injection including 0.1% polysorbat 80) or placebo (replenished with sodium chloride 0.9%). Additionally, one platelet concentrate provided by the blood bank of the University Hospital of Basel will be ordered. Platelet components are either single donor apheresis or buffy-coat pooled platelet components, pathogen-reduced and containing at least 2.4 × 10e11 platelets per unit.

It is recommended to administer 20–50 IE of Wilate® per kg/BW in vWF patients with acute bleeding. The dosage of Wilate® in this trial will be 2000 IU of vWF and 2000 IU factor VIII each in combination with one or two platelet concentrates. The medication (Wilate® or placebo and platelet concentrates) will be administered intravenously, because this is the only possible route of administration of Wilate® and platelets. The objective of this treatment is to stop the haemorrhage by administering the intervention drug once. Due to the long half-time of the study drug, a single administration will be sufficient for the 48-h observation period, also in cases where further thrombocyte transfusions are needed.

To make sure the study drug will be distributed within the body, a minimum waiting period of 10 min should be met whenever possible and depending on the clinical picture before platelet concentrate transfusion (usually this is the case as platelet concentrates have to be ordered in most situations). A waiting period of 20–30 min would be ideal. Thereafter, the clinical situation should be reassessed and the decision to transfuse a second platelet concentrate be taken after careful consideration.

### Control intervention

The control group will be given placebo together with platelets. Our placebo will be provided by the hospital pharmacy of the University Hospital of Basel. The placebo will be sodium chloride 0.9% from Fresenius. The carton contains two Mini-Plasco with 10 ml each. An unblinded member of the ICU team—independent from the study team—will prepare either Wilate® or placebo in a syringe as described in the instructions, which will be delivered with the kit.

The blinding of the trial medication will be a carton, which includes either Wilate® 2× 1000 UI or placebo (sodium chloride 0.9%, 2 × 10 ml).

## Outcome measures

### Primary outcome measure

The primary outcome measure is the number of fresh frozen plasma (FFP) and red blood cell (RBC) transfusions according to groups.

### Secondary outcome measures

Secondary outcomes will consist of the following:

1. Number of platelet concentrates transfused within the first 48 h after treatment of study medication
2. Quantity of blood loss in the first 48 h after treatment with the study medication
3. Length of stay in ICU and hospital
4. Number of revision surgeries or secondary interventions (e.g. re-bronchoscopy, re-gastroscopy) for haemorrhage control
5. Mortality in ICU, hospital, 30-day and 1-year

### Other outcome measures

Additional outcomes include the following:

1. Platelet function analysis as assessed by VerifyNow ® ADP test
2. Amount of administered coagulation factors: 4-factor prothrombin complex concentrate, calcium, tranexamic acid, fibrinogen concentrate, coagulation factor XIII concentrate, vitamin K, antithrombin concentrate and recombinant coagulation factor VIIa
3. Estimate of costs for blood transfusion products during ICU stay (see Supplementary Table 1a and b)
4. Thromboembolic complications

### Safety outcome measures

#### Adverse events

An adverse event (AE) is any untoward medical incident in a patient or a clinical investigation participant administered a pharmaceutical product and which does not necessarily have a causal relationship with the study procedure. An AE can therefore be any unfavourable and unintended sign (including an abnormal laboratory finding), symptom or disease temporally associated with the use of a medicinal (investigational) product, whether or not related to the medicinal (investigational) product. [ICH E6 1.2] AEs are therefore difficult to detect in critically ill patients since the physical state leading to ICU admission itself may already lead to severe complications and further deterioration of physical and mental health.

ICU staff will be instructed to pay attention to allergic reactions like exanthema, flushing, urticaria, stridor, bronchospasm, shock and shortness of breath. These observations will be recorded together with the time of onset, duration, cessation, action that were taken, assessment of intensity and its relation to the study treatment. All adverse events will be reported to investigators and recorded by them on the participating patient’s data sheet during their following visits. Other extraordinary or questionable side effects will also be recorded. The occurrence of thromboembolic events will also be registered until the participating patients are discharged from the hospital.

Serious adverse events (SAEs) are classified as any medical occurrence that results in death, is life-threatening, requires prolongation of existing hospitalisation, or results in persistent or significant disability/incapacity. SAE reporting is required from the responsible Swiss authorities. The third criterion from the SAE definition will be excluded in our trial, as the occurrence of severe bleeding generally leads to prolongation of ICU and/or hospital stay.

A serious unexpected adverse drug reaction (SUSAR) indicates an adverse drug reaction that is of a nature or a severity inconsistent with the applicable product information. Transfusion-related side effects will therefore not be declared as SUSAR.

#### Laboratory parameters

Blood counts and global tests for coagulation, clinical chemistry biomarkers, vWF and FVIII levels and haemoglobin concentration will be analysed. Blood samples will be drawn during regular time points on the intensive care unit (e.g. 6 a.m.) except for the global platelet function analysis (VerifyNow®), which will be analysed right before the first study-specific platelet transfusion and 30–60 min after administration of the study drug and platelets.

#### Vital signs

Vital signs are continually measured at the ICU and recorded in the clinical IT system. The vital parameters include blood pressure, pulse, SpO_2_, ScvO_2_, SvO_2_ and respiratory rate. Also, the lowest temperature measured before ICU admission and again during the 2-day observation period and the fluid balance will be recorded.

## Definitions/conditions

### Inclusion criteria

Adult medical and surgical patients admitted to the ICU who need platelet transfusion with or without severe bleeding (i.e. needing transfusion of at least 2 ECs) will be included.

### Exclusion criteria

#### vWF/VIII concentrate given before study inclusion

Patients receiving factor VIII/vWF concentrate within 48 h (roughly four half-lives of factor VIII/vWF) before inclusion in the study (Haemate ®, Wilate ®) will not be included in the study as data collected from these patients might jeopardise the results.

#### Pregnancy/breastfeeding

Due to alterations in the overall coagulation abilities during pregnancy and lack of experience regarding the treatment during pregnancy and breastfeeding, these patients will be excluded. Moreover, postpartum haemorrhage is mostly managed following specific treatment protocols.

#### Participation in another study with investigational product

For security reasons, these patients will not be included in our study.

#### Overt DIC

Overt DIC for any reason (including trauma-induced DIC) will lead to the affection of the blood’s ability to clot and stop bleeding. Therefore, these patients will also be excluded from our study.

#### Pre-existing thrombocyte/vWF disorders

Patients suffering from innate or acquired platelet disorder (i.e. HIT, TTP, HUS, ITP, sepsis, haemato-oncological disease, other hereditary thrombocytopathies)—with the exception of prior treatment with single or dual antiplatelet agents—or disease affecting vWF (i.e. von Willebrand disease, haemophilia A) will be excluded from the study in order to be able to reliably assess the effect of vWF when added to platelets.

Hypersensitivity to the active substances is defined as a known allergy to vWF or a substance contained in the placebo.

### Primary outcome measure

#### Number of transfusions

This is the number of blood products (fresh frozen plasma (FFP), RBCs, platelet concentrates) transfused within the first 48 h after study allocation in both groups (Tables 2 and 3).

**Table 2:** Baseline data assessed upon study inclusion (data prior/upon admission to ICU) to be documented *before* administration of the study drug

	***Metavision®***	***RedCap®***
Informed consent or statement of an independent physician		X
Randomisation, assignment of the group		X
Demographics^a^	X	
Admission diagnosis		X
Past medical history		X
Relevant medication prior to bleeding (1 month)^b^		X
Type of admission (medical, surgical)		X
SAPS II	X	
SOFA score	X	
Type of surgery (if applicable)		X
Cause of bleeding		X
Reason for thrombocyte transfusion		X
Amount of blood loss prior to ICU admission	X	
Vital parameters^c^	X	
Lowest temperature measured^d^	X	
Lowest temperature measured		
Fluid balance^e^	X	
Full blood count and chemogram^f^	X	
Coagulation laboratory^g^	X	
Platelets function analysis (VerifyNow® ADP test) *before* administration of the study drug		X
Number of given RBCs	X	
Number of given FFPs	X	
Number of given platelet concentrates	X	
Amount of prothromblex	X	
Amount of tranexamic acid	X	
Amount of fibrinogen	X	
Name/amount of other given coagulation products^h^	X	
Transfusion-related side effects (AE, SAE)	X	
Thromboembolic events	X	

^a^Age, sex, BMI, height, social security number (identification log only)

^b^NOACs, antiplatelet agents and oral anticoagulation such as Marcumar® (phenprocoumon), Eliquis® (apixaban), Lixiana® (edoxaban), Xarelto® (rivaroxabam), Pradaxa® (dabigatran), Aspirin ® (ASS) and Plavix® (clopidogrel)

^c^Blood pressure, pulse, SpO_2_, ScvO_2_, SvO_2_ and respiratory rate

^d^Lowest temperature measured before ICU admission

^e^In surgical patients from anaesthesia induction until ICU admission; non-surgical patients: fluid balance (if available) upon ICU admission

^f^Hemoglobin, leucocytes, erythrocites, haematocrit, mean corpuscular volume (MCV), mean corpuscular haemoglobin (MCH), mean corpuscular haemoglobin concentration (MCHC), red cell distribution width (RDW), thrombocytes, quick, international normalised ratio (INR), activated partial thromboplastin time (APTT), thrombin time 1 (TZ1), fibrinogen and β-subunit of human chorionic gonadotropin (βHCG) in female study participants of reproductive age < 45 years

^g^Including von Willebrand factor ristocetin cofactor (VWF:Rco); factors II, V, and VII; INR; and platelets count and level of thrombin

^h^Beriplex, Cyklokapron, Konakion, Kyberin, Fibrogammin, NovoSeven, ionised calcium, etc. (see Additional file 1: Table S1)

**Table 3:** ICU follow-up data to be assessed within the 48-hour study period beginning at study drug administration

	***Metavision®***	RedCap®
Administration of study medication (or placebo)		
Platelet function analysis (VerifyNow® ADP test) 30–60 min after administration of study drug		X
Check if informed consent is possible		X
Amount of blood loss since drug administration		X
Cause of bleeding (if occurred)		X
SOFA score	X	
Vital parameters^a^	X	
Lowest temperature measured^b^		X
Acidosis occurred (pH < 7.35)		X
Duration of acidosis		X
Full blood count^c^	X	
Coagulation laboratory^d^	X	
ROTEM	X	
Level of vWF and FVIII	X	
Haemoglobin concentration	X	
Fluid balance^e^	X	
Number of given RBCs	X	
Number of given FFPs	X	
Number of given platelet concentrates	X	
Amount of Prothromblex after study intervention	X	
Amount of tranexam acid after study intervention	X	
Amount of fibrinogen after study intervention	X	
Name/amount of other given coagulation products^f^	X	
Number of revision surgery for haemorrhage control		X
Transfusion-related side effects (AE, SAE, see 3.7)^g^		X
Thromboembolic events		X
Any recurrent bleeding such as cerebral haemorrhage, DIC		X

^a^Blood pressure, pulse, SpO_2_, ScvO_2_, SvO_2_ and respiratory rate

^b^Lowest temperature measured since ICU admission

^c^Hemoglobin, leucocytes, erythrocites, haematocrit, mean corpuscular volume (MCV), mean corpuscular haemoglobin (MCH), mean corpuscular haemoglobin concentration (MCHC), red cell distribution width (RDW), thrombocytes, quick, international normalised ratio (INR), activated partial thromboplastin time (APTT), thrombin time 1 (TZ1), and fibrinogen^d^ including factors II, V and VII; INR; platelets count; and level of thrombin

^e^Since ICU admission until 48 h after the study drug administration

^f^Beriplex, Cyklokapron, Konakion, Kyberin, Fibrogammin, NovoSeven, ionised calcium, etc. (see above)

^g^48-h observation period

### Secondary outcome measures

#### Number of platelet transfusions

This is the number of platelets transfused within the first 48 h after study allocation in both groups (Tables 2 and 3).

#### Quantity of blood loss

To calculate total blood loss during the first 48 h after the study, drug administration amount into drains and during surgery will be assessed and added up assessing chest tube drainage systems, cardiotomy reservoirs, drainages, suction units and rags, which will be measured and documented however possible. We will also document whether any further blood loss occurred that could not be measured over drainage (e.g. ‘open blood loss’ into surgical drains; Table 1).

#### Other secondary outcomes

Secondary outcome variables like ICU mortality, ICU length of stay, hospital mortality, hospital length of stay, number of bleeding events and number of revision surgeries/re-interventions for haemorrhage control are part of the routine clinical assessment for all hospitalised patients and will be documented accordingly. Patients or their family members will be contacted to assess the rehospitalisation rate, as well as 30-day and 1-year mortality.

### Other outcome measures

#### Platelet function analysis

Before the administration of the study drug and 30–60 min after administration of the study drug, platelet function analysis (VerifyNow® ADP test) will be performed to evaluate the possible improvement of platelet function by timely adding vWF to platelets.

#### Amount and costs of administered coagulation factors

Assessing the amount of administered coagulation factors/blood coagulation vitamins (i.e. 4-factor prothrombin complex concentrate, calcium, tranexamic acid, fibrinogen, coagulation factor XIII concentrate, vitamin K, antithrombin III, recombinant coagulation factor VIIa) will be ensured through documenting the administrated medications during the ICU stay. In addition, the estimated costs for the blood transfusion products will be registered (Tables 2 and 3; Additional file 1: Table S1).

## Reoperations and/or secondary interventions

Any reoperations or secondary interventions (e.g. bronchoscopy or gastroscopy) will be documented.

## Thromboembolic complications

Any thromboembolic events (e.g. venous thromboembolism, pulmonary embolus, stroke, myocardial infaction) occurring during the study period will be recorded.

## Additional bleeding events

Any bleeding events without the need for reoperation and/or secondary intervention requiring any type of additional transfusion (including administration of coagulation factors) will also be registered.

## Specific assessments

### VerifyNow® ADP test

The VerifyNow® assay (Accriva Diagnostics, San Diego, CA) is a turbidometry-based optical detection system that assesses the antiplatelet effect of aspirin and P2Y12 receptor inhibitors in the whole blood, similar to the light transmission aggregometry (LTA) technique [8]. Agonist-stimulated platelets bind to fibrinogen-coated polystyrene beads leading to aggregation and changes in light transmission. The test is performed using citrated whole blood with the addition of different activators including lyophilised ADP and prostaglandin E1. The result is shown as platelet reaction units (PRU) calculated from changes in light transmission. While VerifyNow® has been extensively studied among cardiology patients, mainly in North America, and is well established for these patients, experiences in the perioperative and ICU setting are limited.

In Switzerland, the VerifyNow® is distributed by Axon Lab AG, Baden-Dättwil. No specific handling of blood samples is required for VerifyNow®. After blood sampling in specific blood tubes, they will be placed into the device and the result of the test is provided on the screen.

### vWF:Rco test

Laboratory testing of the VWF ristocetin cofactor (vWF:RCo) will identify vWF activity by quantitative assessment of VWF protein adhesion to platelets or other particles and subsequent detection of the adhered VWF which is facilitated by the inclusion of ristocetin [9].

### Assessment of study drug side effects

Study drug side effects will be assessed as outlined in the ‘Safety outcome measures’ section.

### Assessments documented electronically

Either in our electronic case report form or due to direct data export from our patient data management systems (PDMS: Metavision ®, ISMed ®), we will document information listed in the tables and mentioned follow-up data.

## Randomisation

The trial staff will have permanent access to the electronic case report form where patients are screened and randomised to one of the study arms. Randomisation will be performed via the electronic case report form using REDCap® after checking the eligibility of the patient. A unique patient identification code will be assigned to every screened patient without possible inference to patient identity.

To achieve a balance in the participants in the two trial groups for a small sample size, we plan to perform the so-called block randomisation. The same principle will be performed for the randomisation of the study pack’s serial number. There will be no special criteria for discontinuing or modifying allocated interventions.

## Adherence to intervention protocols

Each time a platelet concentrate is prescribed, a red flag will appear in our data monitoring system. The vWF kit from Basel (HemosIL^2^) will be used to measure the vWF activity (vWF:Ac). The vWF:Ac is measured at four individual times per patient: baseline, 30 min after the thrombocyte concentrate administration has ended, the following day at 6 AM and the day after at 6 AM.

Because the study medication is only a few millilitres and should be given at least 5 min before giving platelets, handling it only for a few minutes will be enough to get it to room temperature, so the study medication will be kept in the fridge until randomisation.

## Blinding

The patient and the treating medical team will be blinded for the treatment. Wilate® and placebo are both provided with a syringe for drawing up the solution. Furthermore, the carton of Wilate® contains 2 Mix2Vial adapter (one for each vial), which is required for the preparation of Wilate®. Wilate® 1000 IU or placebo (sodium chloride 0.9% Fresenius) will be packed in identical-looking opaque cartons. The preparation occurs by unblinded site personnel.

The randomisation list will be maintained with a study-independent chief consultant and will be kept in a secured place in the ICU. To provide all-time immediate emergency unblinding, the storage location will be communicated to the research team. In case emergency unblinding is needed, a member of the study team will ask a study-independent physician to consult the randomisation list.

After the inclusion of all planned participants, the blinded data will be transferred to the responsible statistician for analysis and processing. A blinded analysis is planned after the recruitment of all 120 patients to assess the pre-defined effect size. In case of SAE, the patient will be unblinded for the trial arm.

## Patient information and informed consent

The patients do not always have the capacity to give their consent for the study in advance or at all. In this study, the included patients will mostly be emergency cases. For this reason, an independent auditing physician will declare the patient’s suitability for trial participation in the patient’s name as his representative. As such, the independent physician safeguards the participant’s interest and ensures proper medical care. The signed document of the independent physician is the prevailing condition for the inclusion of the patient in our study.

Depending on the general condition of the patient, the investigators will explain to each participant the nature of the study, its purpose, the procedures involved, the expected duration, the potential risks and benefits and any discomfort it may entail upon enrolment. Each participant will be informed that participation in the study is voluntary and that he/she may withdraw from the study at any time and that withdrawal of consent will not affect his/her subsequent medical assistance and treatment.

If anyhow possible, the participant must be informed that his/her medical records may be examined by authorised individuals other than their treating physician.

All study participants will be provided a participant information sheet describing the study and providing sufficient information for the participant to make an informed decision about their participation in the study.

There will also be a consent form for the participant’s next of kin. The patient and next of kin information sheets and the consent forms will be submitted to the CEC to be reviewed and approved. Formal consent of a participant’s next of kin will be sought in non-emergency cases when the participant will be unable to give consent at any time, and if the next of kin is present or reachable over the phone, using the approved consent form. Consent of next of kin in non-emergency cases may be sought even after the inclusion of the patient. In case of ex-post-study withdrawal by the next of kin, patient data will be destroyed and therefore not be used for publication upon the next of kin’s wish.

In case the participant is able to give consent, he or she should read and consider the statement before signing and dating the informed consent form and should be given a copy of the signed document. The consent form must also be signed and dated by the investigator (or his designee) at the same time as the participant sign, and it will be retained as part of the study records.

Equally, the next of kin should read and consider the statement before signing and dating the informed consent form and should be given a copy of the signed document. The consent form must also be signed and dated by the investigator (or his designee) and it will be retained as part of the study records.

After recovery, the patient will be informed about his participation in the trial and he/she will have the possibility to withdraw his data from the study. On the consent form, participants will be asked if they agree to the use of their data should they choose to withdraw from the trial, since this trial does involve study-specific collecting blood specimens for the VerifyNow® ADP test and the vWF:Rco test. In case of ex-post-study withdrawal, patient data will be destroyed and therefore not be used for publication upon the patient’s wish.

If the participant dies and could not give consent or no next of kin could be contacted for consent (i.e. consent from an independent physician only), recorded data will still be used as the subgroup of patients dying after haemorrhage is highly relevant for data analysis and interpretation. Moreover, as these are critically ill patients, death unfortunately is not a rare event in the ICU.

The investigator affirms and upholds the principle of the participant’s right to privacy and that they shall comply with applicable privacy laws. Especially, the anonymity of the participants shall be guaranteed when presenting the data at scientific meetings or publishing them in scientific journals. Individual subject medical information obtained as a result of this study is considered confidential and disclosure to third parties is prohibited. Subject confidentiality will be further ensured by utilising subject identification code numbers. As indicated in the informed consent forms, there will be no compensation for trial participation.

For data verification purposes, authorised representatives of the sponsor, a competent authority (e.g. Swissmedic) or an ethics committee may require direct access to parts of the medical records relevant to the study, including participants’ medical history.

## Safety

During the entire duration of the study, all adverse events (AE) and all serious adverse events (SAEs) are collected, fully investigated and documented in source documents and case report forms (CRF). Study duration encompassed the time from when the participant signs the informed consent until the last protocol-specific procedure has been completed, including a safety follow-up period.

Post-trial management will not differ from management during the study period and patients will be followed as described in the ‘Study period overview’ section.

An individual subject will be excluded from the study in case of the following:

- Withdrawal of consent by the independent physician or next of kin
- An adverse event that in the opinion of the sponsor contraindicates further measuring (emergency setting)

### Serious adverse reactions

The occurrence of serious adverse events (SAEs) will be assessed during every shift based on the bedside visit and results of vital and laboratory parameters and will be recorded daily on the electronic case report form.

All changes in research activity and unanticipated problems will be reported to the competent Ethics Committee by the sponsor and the principal investigator. An SAE or a serious unexpected adverse drug reaction (SUSAR) must be reported within 7 days if fatal, otherwise within 15 days. The sponsor will provide an annual safety report.

### Relevant concomitant care needs

Since co-administration of Wilate ® to platelets until today is neither part of transfusion guidelines nor part of the indications of Wilate ® implementing platelets with placebo or platelets with Wilate ® will not require alteration to usual care pathways (including use of any medication) for emergency treatment for patients, and these will continue for both trial arms.

## Patient withdrawal

Patients withdrawn (excluding ex-post-study withdrawal) from the study and subjects who could not be followed over the intended period and for all designated points of assessments, regardless of reason will be included in the intention-to-treat analysis. Unless consent for follow-up is withdrawn, subjects discontinued before closeout will be followed for the full study period, and all laboratory and clinical evaluations will occur as defined in the protocol. We can guarantee that the measurements will by no means delay therapy.

## Statistics

Detailed methodology for summaries and statistical analyses of the data collected in this study will be documented in a statistical analysis plan, which will be finalised before database closure and will be under version control by the statistician responsible for the Will-Plate study.

### Hypothesis

Patients with clinical bleeding and an indication for platelet transfusion in the ICU setting have a significant lower number of further blood products (EC, TC, FFP) transfused within the first 48 h after treatment with Wilate® compared to transfusion alone.

### Determination of sample size

A total of 120 (each group 60) patients are needed to detect a 50% reduction of the number of blood products subsequently transfused within two days in patients with Wilate® compared to placebo. The sample size is calculated for a power of 80 % and a significance level of 5% for an estimate of the treatment effect evaluated with a negative binomial regression. To improve the precision of the treatment effect, the following covariates will be included: blood loss, number of blood products transfused within 12 h before administration of the study drug, anticoagulants administered 7 days before, the site of bleeding and the level of thrombocytes at baseline. The sample size calculation is based on a simulation applying resampling techniques on historical clinical data of 150 USB patients that meet the inclusion criteria.

### Planned analyses

#### Datasets to be analysed, analysis populations

All analyses are intention-to-treat analyses. All patients included in the trial will be analysed unless informed consent is withdrawn retrospectively. The description of the dataset is described elsewhere.

#### Primary analysis

Evaluation of the effect of Wilate® compared to placebo on the outcome number of blood products (TC, EC, FFP) administered using negative binomial regression. The following covariates will be included: blood loss, number of blood products transfused within 12 h before administration of the study drug, anticoagulants 7 days before, the site of bleeding and the level of thrombocytes at baseline.

#### Secondary analyses

##### Thrombocytes activity

The *T*-test evaluates the difference in the outcome between the two study groups. The outcome is defined as the difference in the platelet reaction units (PRU) measured with the VerifyNow® ADP test before and after the transfusion of TC.

##### Amount of blood loss

The difference in blood loss in ml within 2 days after platelet transfusion between the study groups will be analysed similarly to the primary endpoint (number of blood products administrated).

##### Mortality

Time-to-event analysis (cox proportional hazard regression) evaluates the difference between the study groups, adjusted for age, disease scores (simplified acute physiology score (SAPS II), sequential organ failure assessment (SOFA score)) and reason for ICU admission.

##### Length of hospital stay

To evaluate the difference of length of hospital stay between the study group, a time-to-event analysis (cox proportional hazard regression) with hospital death as a competing risk will be performed adjusted for age, reason for ICU admission and disease scores (SAPS II, SOFA).

#### Interim analyses

A blinded analysis is planned after the recruitment of all 120 patients to assess the pre-defined effect size.

#### Safety analyses

Descriptive analysis and univariate tests will be performed for the number of SAE by severity grade, transfusion-related side effects, recurrent bleeding and the number of thromboembolic events within 48 h after study drug administration.

#### Other outcomes of interest

1. Amount of administered coagulation factors: prothrombin complex concentrate (Beriplex®), calcium, Cyklokapron (tranexamic acid), fibrinogen, factor XIII (Fibrogammin®), phytomenadione (Konakion®), antithrombin (Kybernin®) and eptacog alfa (NovoSeven®)
2. Estimate of costs for transfused blood products during 48 h of follow-up (see Additional file 1: Table S1) by study group

#### Deviation(s) from the original statistical plan

If substantial deviations of the analysis as outlined in these sections are needed for whatever reason, the protocol will be amended. All deviations of the analysis from the protocol or from the detailed analysis plan will be listed and justified in a separate section of the final statistical report.

### Handling of missing data and drop-outs

The number of dropouts and missing data for the primary and secondary endpoints will be low as the necessary information will be captured during the hospital stay. No complicated treatment regime is in place and the observation period is only 2 days for the primary endpoint.

To assess 1-year mortality, a hospital-wide process based on the AHV number (Swiss social security number) to capture the information from the governmental registry has already been implemented. Patients without a valid AHV number will be contacted personally.

In case of missing covariates, we will apply multiple imputation techniques and perform sensitivity analysis to show that missing information does not change the results of this trial in a substantial way. The main results will be reported based on complete cases.

## Data registration

Data will be entered into a web-based electronic case report form or directly exported from the PDMS systems. Paper case report forms will be used in parallel in case of possible technical difficulties.

## Data handling and management

All data from this study will be kept within the Investigator Site File, and only the study team will have access. In case of a patient’s ex-post denial of study participation, the data collected will not be used for publication involving either the corresponding trial or future trials. In such cases, the data will be destroyed. If the patient does not disagree, data collected until the time point of withdrawal will be used for analysis, but no further data will be collected.

The final trial data for this protocol can be supplied on request. All study data will be archived in a designated place on our intensive care unit at the University Hospital of Basel for a minimum of 10 years after study termination or premature termination of the clinical study.

## Monitoring

Monitoring will be provided by the Department of Clinical Research of the University Hospital Basel.

Monitoring will commence with the study initiation visit, followed by regular monitoring visits within time frames that will have to be determined in the monitoring plan. A member of the study team will conduct at least weekly monitoring of eCRF performance.

The source data/documents are accessible to monitors and questions are answered during possible monitoring.

## Ethical justification

Due to the nature of the condition investigated (severe bleeding representing an emergency setting), patients eligible for study participation will not able to give their consent in most cases. As described above, we will seek the patient’s approval for use of collected data for our publication as soon as feasible.

Severe bleeding is a serious condition calling for immediate intervention. Because ICU length of stay is associated with patient morbidity and mortality we chose to investigate a therapeutic approach that might reduce the need for transfusion products and their possible side effects, thus, leading to shorter ICU and hospital stays and overall better outcomes.

By achieving our goal, we may positively influence patient well-being and enhance satisfaction after severe medical conditions and, most importantly, promote a reduction in patient morbidity and mortality. This in turn would have a positive impact on our society and on the economy.

## Enrolment

The study is planned to begin in March 2021 and will continue for a 3-year period including follow-up.

## Study management and organisation

The study will be organised and managed by the research team of the intensive care unit, University Hospital of Basel. Co-enrolment of study participants in other clinical trials without interventional drug use is basically allowed but will have to be discussed among the competing research teams prior to randomisation.

## Insurance

Insurance will be provided by the sponsor through the liability insurance of the University Hospital of Basel. A copy of the certificate is filed in each trial master file and investigator site file.

## Data sharing and publication

The study results will be communicated to the individual patient when he or she regains the capacity to give study consent. During the ongoing study and until publication, there will be no public access to the data. The datasets analysed during the current study and statistical code are available from the corresponding author upon reasonable request, as is the full protocol or other study documents (e.g. informed consent forms). We plan to publish the data in an open-source major peer-reviewed clinical journal.

A public description of the study in German is available on the Swiss National Clinical Trial Portal (SNCTP).

## Timeline

**Table:** 

2020–2021	- Approval from the competent ethics committee and Swissmedic - Study registration - Funding application - Establishment of eCRF - Development of a monitoring plan - Medical staff study training
2021–2024	- Inclusion of 120 patients - Follow-up of 120 patients - Annual safety report
2024	- Data analysis - Writing and submission of the manuscript for publication

## Discussion

The Will-Plate trial aims to collect innovative data on the effect of adding Wilate ® to platelet concentrates in critically ill patients with respective bleeding. There is limited but promising evidence for improvement of platelet function when additional vWF is given, thereby reducing the need for blood products when compared to placebo. In our study, we aim to confirm the superiority of vWF over placebo for the management of severe blood loss based on high-quality data. The results of this study may lead to better algorithms for the treatment of severe bleeding, which could improve clinical care for patients, reduce the burden of family members and protect the patient’s long-term autonomy and health.

## Patient/public involvement

Patients or the public were not involved in the design of our research and will not be involved in the conduct, reporting or dissemination of our research. This study was registered at the Swiss National Clinical Trial Portal (SNCPT) for official discretion.

## Supplementary Information

**Additional file 1: Table S1. a** Estimated costs of transfusion products (pricing according to centre for blood donation). **b** Estimated costs of coagulation factors (pricing according to www.compendium.ch 2020).

## Data Availability

NA.

## Footnotes

1 Sadler et al. [3]: compendium.ch 2016, URL: https://www.compendium.ch/product/1250481-wilate-trockensub-1000-ie-c-solv#MProproducts (Stand 14.04.2020)

2 URL: https://www.ilexmedical.com/files/ILInserts/vonWillebrandFactorActivity.pdf
