# Supplementary material for: Enhancement of the haemostatic effect of platelets in the presence of high normal concentrations of von Willebrand factor for critically ill patients needing platelet transfusion—a protocol for the will-plate randomised controlled trial
Source: Trials. 2023 Jan 20;24:47. doi: 10.1186/s13063-022-06876-8 (PMC9854010; doi:10.1186/s13063-022-06876-8)
Supplement: Supplementary file 1 — Additional file 1: Table S1. a Estimated costs of transfusion products (pricing according to centre for blood donation). b Estimated costs of coagulation factors (pricing according to www.compendium.ch 2020). [file 13063_2022_6876_MOESM1_ESM.docx]

Will-Plate Study - Tables

Supplementary Table 1a – Estimated costs of transfusion products (pricing according to centre for blood donation).

| **Erythrocyte concentrate from whole blood** | |
| --- | --- |
| *Erythrocyte concentrate leukocytes-depleted* | *233.5* |
| **Thrombocyte concentrate, leukocytes-depleted and inactivated from pathogens** | |
| *Thrombocyte concentrate >2.4x1011Tc/E, from Apheresis* | *1281.25* |
| *Thrombocyte concentrate >2.4x1011Tc/E, pooled from Buffy Coat* | *1281.25* |
| *½ Thrombocyte concentrate >2.4x1011Tc/E, from Apheresis* | *720.00* |
| *½ Thrombocyte concentrate >2.4x1011Tc/E, pooled from Buffy Coat* | *720.00* |
| *Additional HLA-Typing* | *276.75* |
| **Plasma** | |
| *Frozen Plasma, virus-inactivated (Octaplas LG)* | *125.00* |
| **Additions** | |
| *Additional Radiation* | *50.00* |

Supplementary Table 1b – Estimated costs of coagulation factors (pricing according to www.compendium.ch 2020)

| *Advate 250* | *280.85* |
| --- | --- |
| *Advate 500* | *519.35* |
| *Advate 1000* | *997.05* |
| *Advate 1500* | *1472.90* |
| *Advate 2000* | *1950.30* |
| *Advate 3000* | *2904.90* |
| *Adynovi 250* | *269.95* |
| *Adynovi 500* | *498.90* |
| *Adynovi 1000* | *956.85* |
| *Adynovi 2000* | *1872.60* |
| *Alprolix 250* | *445.45* |
| *Alprolix 500* | *849.90* |
| *Alprolix 1000* | *1658.80* |
| *Alprolix 2000* | *3276.65* |
| *Alprolix 3000* | *4894.45* |
| *Baxalta 600* | *509.40* |
| *Benefix 250* | *269.00* |
| *Benefix 500* | *497.45* |
| *Benefix 1000* | *953.40* |
| *Benefix 2000* | *1865.85* |
| *Benefix 3000* | *2717.75* |
| *Beriate 500* | *481.60* |
| *Beriate 1000* | *922.05* |
| *Beriplex P/N 500* | *326.30* |
| *Beriplex P/N 1000* | *611.60* |
| *Cyklokapron (tranexamic acid) 500* | *26.56* |
| *Elocta 250* | *294.35* |
| *Elocta 500* | *547.70* |
| *Elocta 1000* | *1054.40* |
| *Elocta 1500* | *1561.10* |
| *Elocta 2000* | *2067.75* |
| *Elocta 3000* | *3081.15* |
| *Esperoct 500* | *527.90* |
| *Esperoct 1000* | *1014.75* |
| *Esperoct 1500* | *1501.65* |
| *Esperoct 2000* | *1988.50* |
| *Esperoct 3000* | *2962.25* |
| *Fibrogammin 250* | *216.50* |
| *Fibrogammin 1250* | *916.85* |
| *Fibryga 1* | *498.35* |
| *Haemocomplettan P1* | *446.40* |
| *Haemocomplettan P2* | *851.80* |
| *Haemoctin 250* | *247.35* |
| *Haemoctin 500* | *516.25* |
| *Haemoctin 1000* | *991.5* |
| *Immunine STIM 600* | *527.05* |
| *Immunine STIM 1000* | *1012.30* |
| *Jivi 500* | *640.55* |
| *Jivi 1000* | *1240.15* |
| *Jivi 2000* | *2439.25* |
| *Jivi 3000* | *3638.40* |
| *Konakion (Phytomenadione) 10* | *14.25* |
| *Konakion (Phytomenadione) 2 (Pediatric)* | *19.20* |
| *Kovaltry 250* | *306.80* |
| *Kovaltry 500* | *572.60* |
| *Kovaltry 1000* | *1103.55* |
| *Kovaltry 2000* | *2160.75* |
| *Kybernin (Antithrombin III) 500 (Octapharma AG)* | *386.05* |
| *Kybernin (Antithrombin III) 500 (CSL-Behring AG)* | *322.10* |
| *Kybernin (Antithrombin III) 1000* | *524.55* |
| *novoEight 250* | *257.75* |
| *novoEight 500* | *442.90* |
| *novoEight 1000* | *844.80* |
| *novoEight 1500* | *1246.70* |
| *novoEight 2000* | *1648.60* |
| *novoEight 3000* | *2452.40* |
| *novoSeven (eptagogAlfa)1* | *907.55* |
| *novoSeven (eptagogAlfa) 2* | *1774.15* |
| *novoSeven (eptagogAlfa) 5* | *4370.80* |
| *Nuwiq 250* | *276.05* |
| *Nuwiq 500* | *509.70* |
| *Nuwiq 1000* | *976.90* |
| *Nuwiq 2000* | *1901.60* |
| *Octanate 250* | *259.80* |
| *Octanate 500* | *453.65* |
| *Octanate 1000* | *866.10* |
| *Octaplex 500* | *344.05* |
| *Octaplex 1000* | *647.10* |
| *Prothromplex 600* | *369.95* |
| *Refacto 250* | *263.05* |
| *Refacto 500* | *485.05* |
| *Refacto 1000* | *928.65* |
| *Refacto 2000* | *1816.30* |
| *Refacto 3000* | *2703.95* |
| *Refixia 500* | *988.35* |
| *Refixia 1000* | *1935.65* |
| *Refixia 2000* | *3830.30* |
| *Willfact 1000* | *875.40* |
